# Ambient Air Pollution and Depressive Symptoms in Older Adults: Wellenius et al. Respond

**DOI:** 10.1289/ehp.1409657R

**Published:** 2015-05-01

**Authors:** Gregory A. Wellenius, Petros Koutrakis, Yi Wang

**Affiliations:** 1 Department of Epidemiology, Brown University School of Public Health, Providence, Rhode Island, USA; 2 Department of Environmental Health, Harvard T.H. Chan School of Public Health, Boston, Massachusetts, USA; 3 Department of Environmental Health, Indiana University Richard M. Fairbanks School of Public Health, Indianapolis, Indiana, USA

We thank Gao et al. (2015) for their interest in our recent publication examining the association between ambient air pollution and depressive symptoms in a cohort of Boston-area elderly participants (Wang et al. 2014).

Gao et al. (2015) suggest that the null results we found are due to excessive exposure measurement error stemming from the use of pollutant concentrations measured at a single stationary monitoring site. In this investigation we examined associations with short-term exposure to ambient fine particulate matter mass, sulfate, black carbon, and ultrafine particles measured at the Harvard–U.S. Environmental Protection Agency Supersite, which is located < 20 km from the participants’ homes. Particle measurements from this monitoring site have been shown to be strong proxies for personal exposure to particles of ambient origin (Brown et al. 2009) and have been used in hundreds of prior studies. Nonetheless, as we acknowledged in our article, exposure misclassification likely resulted in wider confidence intervals for our effect estimates, but is not expected to have biased our results (Zeger et al. 2000). We also failed to find evidence of an association with long-term exposure to traffic pollution based on both residential proximity to major roadways and residential black carbon levels predicted by a spatiotemporal model (Gryparis et al. 2009). Both of these exposure metrics have been used in a large number of air pollution health effects studies in the Greater Boston area, most of which have found associations with a large spectrum of health outcomes (Hart et al. 2014; Lue et al. 2013; Suglia et al. 2008; Wellenius et al. 2012; Wilker et al. 2013).

Gao et al. (2015) further suggest that our null results might be due to excessive misclassification of the outcome because we assessed the presence of depressive symptoms using the Revised Center for Epidemiological Studies Depression Scale (CESD-R) (Eaton et al. 2004) rather than using a scale specifically designed for the elderly or relying on clinical diagnoses of depression. The CESD-R has been validated in the general population (Van Dam and Earleywine 2011), and the Center for Epidemiological Studies—Depression Scale, on which the CESD-R is based, has been validated (Radloff 1977) and used extensively to study depressive symptoms in the elderly. Nonetheless, psychometric properties clearly differ across instruments, and the use of different instruments certainly could contribute to the heterogeneity observed across studies. Moreover, as we discuss in our paper, the CESD-R assesses the presence of depressive symptoms within the preceding 2 weeks rather than depression episodes requiring clinical attention or the presence of clinically diagnosed depression.

In their letter, Gao et al. (2015) criticize our decision not to adjust for prevalent cardiovascular disease (CVD) in our primary analyses, suggesting that we should have adjusted for it because *1*) air pollution is believed to cause CVD, and *2*) there is a well-documented association between depression and CVD. However, CVD is a downstream consequence of exposure to air pollution, and therefore, adjusting for it in analyses of air pollution health effects requires caution and appropriate caveats (Hernan et al. 2002). Nonetheless, in our paper we presented sensitivity analyses that were additionally adjusted for body mass index, physical activity, alcohol consumption, smoking, diabetes mellitus, hypertension, and hyperlipidermia, and showed that the results were not materially different. Thus, it does not seem likely that our null results are due to lack of adjustment for CVD or its risk factors.

Finally, Gao et al. (2015) suggest that our results stand in contrast to those of “most previous studies.” This may be true, but it is worth noting that there are very few other studies available for direct comparison, and thus this remains very much an open research question. Additional studies in diverse populations are clearly needed to confirm or refute the presence of an association between air pollution and depressive symptoms.
